# Preserving Vaccine Virus

**Published:** 1871-11

**Authors:** 


*Preserving Vaccine Virus.*—Dr. D. B. Hillis, in the "Pharmacist," gives a method of preserving vaccine virus between layers of dental red rubber, and remarks as follows in reply to an inquiry on the subject:

"In reply to your note of inquiry as to the efficiency of the plan of preserving vaccine virus between layers of the red rubber used by dentists, I state that for several years past I have used no new matter, but have, for the sake of experiment, drawn from the stock originally put up as described. I have always found it effective, though no care whatever has been taken as regards temperature. Last week I succesfully vaccinated a patient from a crust which had been lying about my office for *twenty-seven* months."

